# Physiological and Metabolic Responses to Water Restriction in Ewes Under Semi-Arid Conditions

**DOI:** 10.3390/vetsci12090790

**Published:** 2025-08-22

**Authors:** Claudenilde de Jesus Pinheiro Costa, Gherman Garcia Leal de Araújo, André Luiz Rodrigues Magalhães, Alberício Pereira de Andrade, Silvia Helena Nogueira Turco, Maria Helena Tavares de Matos, Diego César Nunes da Silva, Cleyton de Almeida Araújo, Roberta de Lima Valença, Thieres George Freire da Silva, Fleming Sena Campos, Glayciane Costa Gois

**Affiliations:** 1 Universidade Federal do Agreste de Pernambuco, Garanhuns 55292-270, Brazil; claudenildepinheiro@gmail.com (C.d.J.P.C.); andre.magalhaes@ufape.edu.br (A.L.R.M.); albericio.andrade@ufape.edu.br (A.P.d.A.); 2 Embrapa Semi-Árido, Petrolina 56302-970, Brazil; gherman.araujo@embrapa.br; 3 Universidade Federal do Vale do São Francisco, Petrolina 56300-000, Brazil; silvia.turco@univasf.edu.br (S.H.N.T.); helena.matos@univasf.edu.br (M.H.T.d.M.); diego.nunes@univasf.edu.br (D.C.N.d.S.); cleyton.araujo@univasf.edu.br (C.d.A.A.); 4 Universidade Federal do Espirito Santo, Alegre 29500-000, Brazil; roberta.valenca@ufes.br; 5 Universidade Federal Rural de Pernambuco, Serra Talhada 56909-535, Brazil; thieres.silva@ufrpe.br; 6 Universidade Estadual do Sudoeste da Bahia, Itapetinga 45700-000, Brazil; fleming.campos@uesb.edu.br; 7 Universidade Federal do Maranhão, Chapadinha 65500-000, Brazil

**Keywords:** dehydration, red blood cells, respiratory rate, small ruminants, total proteins

## Abstract

The aim of this study was to evaluate the effect of different water restrictions on the thermoregulation and blood hematological and metabolite parameters of crossbred Santa Inês ewes in a semi-arid climate. Thirty-two ewes were subjected to four water supply levels (100%, 80%, 60%, and 40%), in a completely randomized design with eight replications. The confinement period lasted 77 days, with 14 days allocated for adaptation. Respiratory rate, heart rate, and rectal temperature exhibited a quadratic response. There was an increase in red blood cells and urea. The enzyme alanine aminotransferase decreased linearly with water restriction. Urinary creatinine decreased along with water supply. Regarding urine color characteristics, all groups showed different colors, ranging from clear to cloudy. For the chemical characteristics of urine, a quadratic effect was observed for pH, with the highest value (8.75) at 60%. An increase was observed in total urine proteins and urobilinogen. Crossbred Santa Inês ewes in a semi-arid climate exhibit physiological adaptations to water supply reduction up to 40%. Following an 80% reduction in water supply, animals exhibit mild dehydration, characterized by increased serum urea levels and decreased alanine aminotransferase activity.

## Simple Summary

Water scarcity in a semi-arid region can affect the thermoregulatory responses of ewes, compromising their physiological balance and productivity. To evaluate this impact, different levels of water were offered to sheep in the Brazilian semi-arid region, simulating conditions of moderate to severe restriction. Ewes submitted to a 40% water supply have higher respiratory and heart rates, indicating greater thermoregulatory effort and stress. These alterations reflect the animals’ attempt to dissipate heat under limited hydration. Additionally, urinary creatinine decreased with reduced water supply to ewes, suggesting altered renal function or muscle metabolism. In contrast, total urine proteins and urobilinogen increased with reduced water supply to ewes, pointing to possible liver stress and changes in protein catabolism. These physiological and metabolic changes highlight the vulnerability of ewes to water restriction and emphasize the importance of adequate hydration, especially in regions subject to prolonged drought.

## 1. Introduction

Dryland regions are characterized by low water availability, high temperatures, high incidence of solar radiation, low humidity, and high rates of skin evaporation [1]. These environmental factors significantly impact the productivity and well-being of sheep in these regions worldwide, particularly concerning water supply, as water plays a fundamental role in animal thermoregulation. Its scarcity or deprivation causes an increase in heat stress [2].

Water is the most important nutrient for the maintenance and performance of small ruminants, contributing to proper digestion, absorption, metabolism, nutrient transport, and waste elimination [3]. Thus, any change in its availability can lead to a direct alteration in animal behavior, as they develop different mechanisms to tolerate dehydration, responding to stress factors with minimal changes in homeostasis [4].

Intermittent water supply is a management practice adopted in confinement systems in semi-arid regions, which can expose small ruminants to water restriction [5]. Previous studies conducted by our research group have shown that lambs raised in semi-arid conditions, with freshwater restriction and competition with irrigation and human consumption, can tolerate intermittent water supplies for 48 h and 72 h [6,7,8] and even ingest water with a high salinity (8320 mg TDS/L; [2,9]). According to Adeniji et al. [10], animals that have access to water once a day or to a percentage of their ad libitum intake, or are subjected to total water deprivation tend to experience a greater sensation of thirst and drink the available water within up to 2 min, remaining without water until the next period when water is provided again. Saini et al. [11] observed that dehydration limits cardiovascular and thermoregulatory responses, resulting in an increase in core temperature due to reduced skin blood flow and sweating rate. Therefore, studies are needed to analyze the effect of water restriction on the thermoregulatory responses of animals to obtain information and knowledge that can aid in decision-making to optimize ewe production in semi-arid climates.

Climate projections indicate that semi-arid regions will be affected by climate change in the coming decades. According to the Intergovernmental Panel on Climate Change [12], there is a projected increase of 12% in global average temperature and a 17% reduction in rainfall by the end of the 21st century. Due to its high climatic risk and the high incidence of socio-economic challenges, the Brazilian semi-arid region is recognized as a critical hotspot of socio-environmental vulnerability [13]. These changes may intensify challenges related to thermal comfort, water balance, and livestock production. In this context, the development of management strategies that enable the understanding and mitigation of the adverse effects of heat stress on ruminants becomes essential.

The ability to tolerate water restriction can be evaluated by observing physiological and biochemical parameters such as heart rate, respiratory rate, and serum or behavioral analyses [14,15,16]. These parameters are simple measurements to assess the health status of confined animals. Mengistu et al. [17], when studying the effects of water restriction at 90%, 80%, 70%, 60%, 50%, and 40% of ad libitum intake over periods of 1 and 2 weeks in goats and sheep, observed that water restriction between 40% and 50% did not alter the animals’ physiological responses during the 2-week period. Yetisgin and Şen [18], in a study on the drought resilience of confined Awassi ewes in a semi-desert environment subjected to 50% water restriction, emphasized that the lack of an adequate water supply for small ruminants can limit their productivity and critically affect physiological responses. These effects include reductions in metabolic rates as a possible energy conservation strategy, decreases in plasma volume due to water being absorbed by tissues, and impairments in feed intake and nutrient utilization, which may negatively impact both fetal and placental development. Therefore, investigating the effects of water restriction in ewes subjected to extended confinement periods may provide valuable and relevant insights into potential future scenarios driven by the risks associated with climate change in the Brazilian semi-arid region.

We hypothesize that increasing levels of water restriction will alter thermoregulatory and metabolic parameters in Santa Inês ewes. Thus, the aim of this study was to evaluate the effect of different water restrictions on the thermoregulation and blood hematological and metabolite parameters of crossbred Santa Inês ewes in a semi-arid climate.

## 2. Materials and Methods

### 2.1. Location

The study was conducted at the UNIVASF, Petrolina-PE, Brazil (9°19′28″ South latitude, 40°33′34″ West longitude, 393 m altitude), during the period from February to April 2020. The region’s climate is classified as BSwh, characterized as a semi-arid climate with an average annual precipitation of approximately 435 mm, average temperatures ranging from 24.5 to 33.8 °C, and relative humidity varying between 69.3% and 73.56% [19].

This research is part of a larger project with a methodology based on the study of productive performance [20], carcass yield and digestive compartments [21], and meat quality [22]. All procedures described in this study were approved by the Ethics Committee of UNIVASF (Process No.: 0002/241017). During the experimental period, the maximum and minimum temperatures were 33.8 °C and 24.5 °C, respectively, with relative humidity (RH) ranging from 65.6% to 73.56%.

### 2.2. Animals, Management, and Study Design

Thirty-two crossbred Santa Inês ewes with an average body weight of 32.2 kg and an average age of 2.5 years were housed in individual pens (1.00 × 1.20 m) distributed in a covered shed equipped with drinkers and feeders. The confinement period lasted for 77 days. Throughout the study, the ewes received the same diet, provided twice daily (9:00 and 15:00). A randomized complete block design was adopted with 4 treatments and 8 replicates. The diet was formulated according to NRC [23] with a roughage:concentrate ratio of 46:54 on a dry matter basis (Table 1).

During the confinement, the ewes had an average dry matter and crude protein intake of 1150 and 0.16 kg/day (100% intake), 1100 and 0.15 kg/day (80%), 1100 and 0.15 kg/day (60%), and 1080 and 0.15 kg/day (40%). This information was presented by Lima et al. [20] in a previous study.

### 2.3. Water Treatment

The treatments consisted of different water supplies: control 100%, 80%, 60%, and 40% of the control group’s consumption (Table 2). Water was provided to the animals daily at 09h00 in buckets with a capacity of 10 L. The water was weighed before and after 24 h. Three buckets containing water were distributed in the shed near the animal cages to determine daily evaporation. Water evaporation was considered when the remaining water was approximately 100 g relative to the amount initially offered. Samples of the water offered to the animals were collected biweekly for physicochemical analysis and showed the following composition: pH (6.98), Ca^2+^ (0.63 mmol/L), Mg^2+^ (0.74 mmol/L), Na^+^ (0.27 mmol/L), K^+^ (0.18 mmol/L), Cl^−^ (0.66 mmol/L), bicarbonates (0.32 mmol/L), sulfates (0.51 mmol/L), total hardness CaCo_3_ (3.44 mg/L), electrical conductivity (0.08 ds/m).

The average water intake values per treatment were 1.79 L/day (100%), 1.41 L/day (78.77%), 1.11 L/day (62.01%), and 0.73 L/day (40.78%).

### 2.4. Environmental Variables

Black globe temperature, relative humidity, and air temperature were recorded every minute using data loggers installed in the confinement shed. With the collected data, the Temperature–Humidity Index (THI) [24] was determined. Thermal comfort/stress ranges were classified according to Silanikove and Koluman [25].

### 2.5. Physiological Parameters

The physiological parameters were measured in three collections conducted on non-consecutive days. Measurements were taken every 3 h (09h00, 12h00, 15h00, 18h00, 21h00, 00h00, 03h00, and 06h00). Respiratory rate (RR, min) was determined by counting flank movements for 20 s [26]. Heart rate (HR, min) was obtained by counting the number of movements in the left thoracic region at the approximate height of the aortic arch for 15 s [26]. The values obtained for RR and HR were multiplied by 4 to obtain the results in variables per minute. Rectal temperature (RT, °C) was measured using a clinical thermometer inserted into the animal’s rectum for two minutes. The thermometer was inserted to a depth of 2 cm, remaining in contact with the mucosa [26].

Sweating rate (SR) was measured in 3 collections conducted on non-consecutive days. Measurements were taken at two times, 09-h and 15-h. The ventilated capsule technique proposed by Maia et al. [27] was adopted. The capsule was connected to a dehumidifier, and ambient air was drawn into a Falcon tube containing silica. The capsule was attached to the animal’s body surface, in the neck and loin regions, for 90 s so that the silica in the capsule could absorb moisture from these regions. After this period, the tubes with silica were removed, sealed, and weighed. The difference in weight before and after the process represents the amount of evaporated water that was absorbed. The sweating rate was determined using the equation:

(1) SR = X × λ /A × T 

where *X* = difference in the weight of the silica before and after the animal assessment, *λ* = latent heat of vaporization of water, *A* = area of the capsule attached to the animals (*A* = 0.002123 m^2^), and *T* = contact time between the capsule and the animal’s surface (*T* = 90 s).

### 2.6. Biochemical and Hematological Parameters

Blood was collected every 15 days before the provision of the diet by jugular vein puncture. Three blood samples were collected in vacuum tubes (Vacutainer^®^), one of which contained 10% EDTA anticoagulant for the hemogram. After collection, the tubes were transported in an insulated box containing ice to the laboratory, where the following biochemical parameters were determined: albumin, creatinine, glucose, urea, total serum proteins (TP), cholesterol, triglycerides, gamma-glutamyl transferase (GGT), aspartate aminotransferase (AST), and alanine aminotransferase (ALT). The analyses were performed using a semi-automatic biochemical analyzer (DKP-620-BI, Prolab, São Paulo, SP, Brazil) with activities quantified by commercial kits (BioSystems^®^, Recife, PE, Brazil). Hematological analyses included mean corpuscular hemoglobin (MCH), white blood cell count (WBC), red blood cell count (RBC), platelet count (PC), hemoglobin count (HGB), hematocrit (HCT), platelet distribution width (PDWC), mean corpuscular volume (MCV), red cell distribution width (RDWC), mean platelet volume (MPV), mean corpuscular hemoglobin concentration (MCHC), and procalcitonin (PCT). The samples were processed using an automatic hematology analyzer (Hematoclin 2.8 Vet—R666 Bioclin, Belo Horizonte, MG, Brazil).

### 2.7. Urine Analysis

Urine was collected daily during the ewe’s spontaneous urination. The urine was collected in plastic bottles. A 10% aliquot of the total urine was stored in labeled plastic containers for analysis. Urinary creatinine and urea were determined using commercial kits (Bioclin^®^, Biomaxlab, Maceió, AL, Brazil). For the evaluation of crystals present in the urine, sediment analysis was determined according to Garcia-Navarro [28], which included the identification of leukocytes and yeasts. Cells were numerically quantified per field, ranging from rare cells to countless. Other structures, such as bacteria and cells, were part of the sediment analysis and were quantified using a cross system, ranging from absent to 3 crosses, according to Santarosa et al. [29].

For physicochemical analyses, the following parameters were evaluated: density, color, appearance, pH, proteins, urobilinogen, and red blood cells. Urine density was determined using a portable refractometer (RFATC200, Benfer, São Paulo, SP, Brazil). The color was visually classified as light yellow, straw yellow, and citron yellow [30]. The appearance was evaluated subjectively and classified as clear or cloudy. The pH was measured with the electrode inserted into the urine sample. The determination of proteins, urobilinogen, and red blood cells in the urine was carried out using reagent strips (Uriquest Plus, Labtest, Lagoa Santa, MG, Brazil).

### 2.8. Statistical Analysis

The data were analyzed by the PROC GLM of SAS University [31] and subjected to analysis of variance and regression at a 5% probability, with the decomposition of the sum of squares of the treatments in contrasts related to linear and quadratic effects, with adjustment of the equations of regression. The following statistical model was used:

(2) Y= μ+ Bi+ Tj+ eij 

where *μ* = overall mean; *B*i = block effect; *T*j = effect of different water supplies; *e*ij = residual error.

The criteria for choosing the regression models were the significance of the parameters estimated by the models and the values of the determination coefficients (R^2^). The standard error of the mean was obtained from the raw data.

## 3. Results

**Figure 1:**
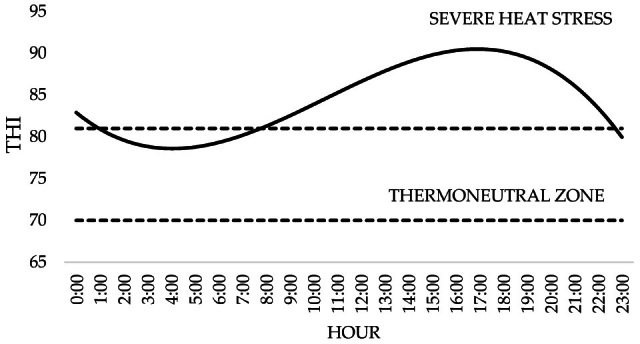
Temperature and Humidity Index (THI) at different times of the day.

During the experimental period, the THI increased starting at 08h00, remaining above 80 until 22h00 (Figure 1). It was observed that after 14h00, the THI reached 90, which continued until 19h00, then declined subsequently (Figure 1).

Water restriction had a quadratic effect on RR (*p* < 0.001), HR (*p* < 0.001), and RT (*p* < 0.001). There was no effect of water on the SR of the neck or loin (*p* > 0.05) (Table 3).

It was observed that the highest respiratory rate was presented by the ewes at 15h00, with 93.81 breaths/min (*p* < 0.001). Between 12h00 and 15h00, the ewes showed the highest heart rate, with values ranging from 111.03 to 107.28 beats/min (*p* < 0.001). Regarding rectal temperature, the highest RT values were measured between 15h00 and 18h00 compared to the RT values measured in the ewes during the period between 00h00 and 06h00 (*p* < 0.005) (Table 4). There was no effect of the different times on the SR of the neck or loin (*p* > 0.05) (Table 4).

It was observed that the highest respiratory rate was presented by the ewes at 15h00, with 93.81 breaths/min (*p* < 0.001). Between 12h00 and 15h00, the ewes showed the highest heart rate, with values ranging from 111.03 to 107.28 beats/min (*p* < 0.001). Regarding rectal temperature, the highest RT values were measured between 15h00 and 18h00 compared to the RT values measured in the ewes during the period between 00h00 and 06h00 (*p* < 0.005). There was no effect of the different times on the SR of the neck or loin (*p* > 0.05) (Table 4).

For the biochemical parameters, it was observed that urea levels increased as the water supply for the ewes was reduced (*p* = 0.002). The opposite was observed for ALT, which decreased with the reduction in the water supply (*p* = 0.037) (Table 5). There was no effect of water restrictions on albumin, creatinine, glucose, TP, cholesterol, triglycerides, AST, or GGT (*p* > 0.05) (Table 5).

Red blood cell count increased with the reduction in the water supply (*p* = 0.022). The other hematological parameters evaluated were not affected by the water treatments (*p* > 0.05) (Table 6).

The reduction in water supply resulted in a decrease in the creatinine concentration in the urine of the ewes (*p* = 0.020). There was no effect of water supply on the urea concentration in the urine (*p* > 0.05). Through sediment analysis, it was observed that ewes that received 80% and 40% of the water supply had leukocytes in their urine (1/8). The highest frequency of crystals (4/8) and yeasts (1/8) was observed in the urine of ewes that received 80% of the water supply (Table 7).

Bacteria were present in almost all urine samples evaluated in each treatment. Ewes that received the control treatment (100% of the water supply) and the treatments with 80% and 60% of the water supply had a bacterial count in their urine between 1 and 3 cells/field (++), while ewes that received 40% of the water supply had a bacterial count in their urine >5 cells/field (+++) (Table 7). Regarding cells, it was observed that ewes that received 100% of the water supply had rare cells in their urine (6/8). Ewes that received 80% of the water supply had a higher moderate cell count (3/8) in their urine, while ewes that received 40% of the water supply had a high cell count (3/8) in their urine (Table 7).

The pH of the urine showed a quadratic effect as the water supply for the ewes was reduced (*p* = 0.034). An increase in the proteins (*p* = 0.039) and urobilinogen (*p* = 0.050) concentrations in the urine was observed with the reduction in the water supply. There was no effect of water restrictions on the density or concentration of red blood cells in the urine (*p* > 0.05) (Table 8).

All samples showed a yellow coloration, ranging from light yellow to citrine yellow for the urine of ewes that received 60% and 40% of the water supply. The urine of ewes that received 100% of the water supply showed a straw yellow and citrine yellow coloration, while the urine of ewes that received 80% of the water supply showed a light yellow and citrine yellow coloration (Table 8). The urine of ewes that received 80% and 60% of the water supply was mainly turbid (5/8), while the urine of ewes that received 100% and 40% of the water supply showed a similar result for appearance (4/8 clear; 4/8 turbid) (Table 8).

## 4. Discussion

The determination of THI is important for identifying environmental comfort and quantifying the thermal stress to which the animal is subjected based on meteorological conditions [32]. According to the results obtained in this study, it was observed that the animals were mainly under conditions of stress (THI between 80 and 85) and extreme stress (THI above 88), according to Silanikove and Koluman [25]. It was noted that the periods with the highest respiratory rates also corresponded to the periods with the highest THI, indicating that temperature and humidity had a direct influence on the stress of the ewe. This behavior was expected, as there is a high correlation between respiratory rate and THI [33].

A higher respiratory rate was observed in the group of ewes that were under water restriction (60% and 40%), indicating that there was heat loss through the upper respiratory system. Water consumption is one of the ways animals exchange heats. When drinking water is consumed, its lower temperature compared to that of the animal promotes heat dissipation, resulting in a reduction in body temperature. Heat is dissipated through latent heat of vaporization through the upper respiratory system, as ewes are panting animals [34]. However, in situations where drinking water is limited, such as in cases of water restriction, the animal seeks other thermoregulatory mechanisms, such as evaporation in the respiratory system, resulting in an increased respiratory rate. This is the first mechanism for panting animals, such as ewes. Panting could be fast and shallow in the beginning then deep and low, so at this stage the animal will lose more water from their body. This mechanism occurs when the inhaled air, in contact with the moisture of the pulmonary alveoli and the walls of the respiratory ducts, causes evaporation, as the exhaled air is almost saturated with water vapor, which contributes to heat loss [35].

In light of the above, it is known that the more panting the animal is, the greater the heat stress it is under. The average respiratory rates were higher in the afternoon period. There was an effect regarding the time of day and respiratory rate for both periods, with an increase from 46 to 67 breaths/minute in the morning period and from 85 to 93 breaths/minute in the afternoon period, which may be related to body temperature and circadian rhythm, as sheep are diurnal animals. This can be explained by water evaporation through the airways, which is the most efficient mechanism for dissipating excess body heat in ewes [36]. Mendes et al. [37] reported that Dorper breed sheep in the Brazilian semi-arid region showed a respiratory rate of 170.75 breaths/minute, observed in the afternoon period, in the shade. This indicates that even in the shade, animals show an increase in respiratory rate.

Heart rate increases as air temperature also increases [38]. Thus, the heart rate of the ewes in this study was influenced both by environmental conditions and by the reduction in water supply, presenting heart rate values above the normal limit considered for the species, which is 70 to 80 beats/minute [39].

The slight increase in rectal temperature in the animals from the treatment with 40% water supply compared to those from the treatments with 80% and 60% can be attributed to the decrease in evaporative cooling due to the lower availability of water to maintain body temperature. De et al. [40], working with water restriction (20% and 40%) in Malpura breed sheep, observed that rectal temperature remained elevated among the groups in the morning and afternoon shifts. In this research, the ewes maintained their body temperature within the normal range (ranging from 38.3 °C to 39.9 °C; [41]), which possibly indicates adaptability to water stress, increasing RR through panting and thus heat dissipation. McManus et al. [42] state that when there is an increase in rectal temperature, it means that the animal may be storing heat, and if there is no dissipation, then heat stress is expressed.

The highest RT, recorded between 12h00 and 21h00, coincided with the peak THI values, with the maximum RT observed at 15h00 (39.20 °C). This aligns with the normal circadian rhythm, indicating that the animal is not retaining heat and is not in a state of hyperthermia. Sheep breeds developed in tropical and semi-arid climates, such as Santa Inês, show greater tolerance to heat stress, and even though RT is affected by high temperatures and reduced water supply, these animals have greater tolerance to changes in RT [33].

The loss of body fluids results from heat dissipation mechanisms (sweating and evapotranspiration) in an attempt to maintain temperature within physiological limits [43]. In this study, we did not observe any effect of different water supplies or evaluation times on sweating rate, suggesting that the reduction in water supply did not influence homeothermy. Possibly, these animals were able to regulate internal temperature without activating sweating, since they are not primarily sweating animals, which is the last defensive mechanism to maintain body temperature. As cutaneous evaporation is an important way for the animal to lose heat to maintain thermal balance [44], it demonstrates an adaptive capacity to the environment and water supplies.

The components of the hemogram evaluated in this study are within the reference values for the ovine species [45]. The increase in the number of red blood cells with the reduction in water supply can be explained by the decrease in blood volume, leading to the concentration of red blood cells [46]. Hematocrit, which is the ratio of red blood cells to serum, is the best indicator of water shortage and could be related to water supply.

The increase in serum urea can be attributed to the hypovolemic state caused by dehydration in the animals. Urea is produced by the liver and excreted by the kidneys, eliminating ammonium ions derived from amino acid metabolism, as well as from ruminal and intestinal microbial activity. According to Getahun et al. [47], part of the ingested protein is converted into ammonia, amino acids, and peptides in the rumen, with ammonia being used for microbial protein synthesis. When the rate of ammonia production exceeds microbial requirements, the excess is absorbed through the ruminal epithelium and converted into urea by the liver. Part of this urea may be recycled, while the remainder enters the bloodstream and is excreted by the kidneys [48]. Casamassima et al. [49] reported that water deficiency increases water reabsorption in the nephron, consequently leading to increased reabsorption of urea. Although this response is part of the physiological adaptation to water restriction, the maintenance of elevated serum urea levels over prolonged periods may impair renal function. Nevertheless, despite the values reported in this study being above the normal physiological range for sheep (17.12 to 42.8 mg/dL [50]), the animals did not show clinical symptoms of renal dysfunction. Supporting our findings, Hamadeh et al. [51], when evaluating the effect of water deprivation on urea concentration in Awassi ewes, observed that serum urea levels increased with water restriction and stated that this increase may be due to reduced glomerular filtration and increased urea reabsorption, thereby improving nitrogen balance in the ewes.

The reduction in the hepatic enzyme ALT suggests the presence of hepatocyte lesions in response to dehydration, as this blood enzyme is considered an indicator of liver health [52], and it is involved in the synthesis of amino acids in the liver [53]. ALT values remained below the reference range (26.0–34.0; [50]), possibly due to water stress. Similar to our results, Akinmoladun et al. [54] and Noureddine et al. [55], when working with small ruminants subjected to water restriction, observed that ALT concentrations were also below the reference value for this enzyme. The authors attributed such an increase to hemoconcentration of the enzyme in question and the animals’ adaptive capacity to water stress.

Creatinine levels can be increased by water restriction [55], a fact not observed in this study. However, despite the decrease in creatinine values with the reduction in water supply, these were considered normal for the ovine species (ranging from 52.45 to 39.93 mg/dL; [56]). The reduction in creatinine content can be explained by the possible reduction in muscle protein catabolism caused by the higher concentration of thyroid hormones resulting from increased thyroid activity due to animal exposure to high ambient temperatures [55].

In the urinary sediment analyses, crystals were present in all groups, with the highest quantity observed in the urine of ewes that received 80% of the water supply. Two restriction groups showed leukocytes, and only one group had yeast in the urine. This behavior can be explained by the fact that water deprivation reduces the appearance of these sediments in ewe’s urine. For bacteria analyses, a large part was absent, and for cell samples, the rarity of cells was classified as normal [28].

All animals presented urine with a yellowish color, which is considered normal for the species [29], varying only in its shade, ranging from light yellow to citrine yellow, and with an aspect between clear and turbid. According to Taffarel et al. [30], the yellowish color of urine is due to the presence of urochromes, while urine turbidity results from the appearance of mucus, bacteria, or shedding cells.

The urine pH ranged from 7.62 to 8.75, in accordance with the normal reference value for ovine urine pH (7.0 to 8.0; [28]). One factor that may have contributed to the increase in urinary pH is evapotranspiration. This mechanism, triggered in an attempt to maintain homeothermy, usually results in an increase in RR, which in turn causes the animal to eliminate a greater amount of CO_2_, promoting alkalosis. In response, the kidneys increase the excretion of HCO_3_^−^ and reduce the excretion of H^+^ ions in an attempt to maintain acid–base balance [57].

Water restriction in small ruminants triggers endocrine responses aimed at maintaining hydroelectrolytic homeostasis and regulating energy metabolism. Among the hormones involved, vasopressin acts on the renal tubules by promoting water reabsorption, reducing urine volume, and increasing its concentration as a strategy for water conservation [58]. The reduction in urine volume observed in the ewes studied here was previously reported by Lima et al. [20], which may also be associated with an increase in Na^+^ concentration due to enhanced aldosterone activity, as this hormone raises electrolyte levels in the kidneys [59]. Aldosterone, secreted by the adrenal cortex in response to activation of the renin–angiotensin–aldosterone system, promotes sodium retention and, secondarily, water retention, thus contributing to the maintenance of plasma volume. In addition, cortisol levels increase as a result of energy reserve mobilization [60]. Li et al. [61] reported that, under water restriction and heat stress, sheep exhibited elevated cortisol and aldosterone secretion to maintain water balance. Thus, future studies evaluating plasma concentrations of vasopressin, aldosterone, and cortisol in ewes subjected to water restriction under confinement for 77 days are necessary and relevant.

The presence of protein was observed in the urine of all animals under water restriction. This fact may be explained by renal filtration, where decreased water intake may have compromised this function, retaining protein due to its high molecular weight [28]. Regarding the presence of urobilinogen, Ferreira et al. [62] mention that values up to 0.10 mg/dL of urobilinogen in urine are considered normal; however, above this value, there is an increase in bilirubin in the urine. Thus, the ewes that received 40% of the water supply showed urobilinogen levels above the recommended, which may indicate a state of dehydration.

Although the results obtained provide relevant information about the effects of water restriction on Santa Inês ewes confined in a semi-arid region, it is important to recognize some limitations that may have influenced the results obtained in this study. For example, the absence of prospective hematobiochemical data restricts the ability to analyze, with greater precision, the temporal trends in the physiological responses of the animals throughout the experimental period. This limitation hinders the identification of metabolic adaptations or physiological disorders that may have occurred progressively during exposure to water restriction.

Additionally, the use of a single diet, characterized by a high mineral salt content, may have possibly caused a water imbalance and affected the urine composition of the animals. The interaction between the mineral intake of the diet and the physiological mechanisms of water retention or excretion may have either enhanced or attenuated some of the observed responses. Thus, future studies are necessary and relevant to monitor the dietary effect and the different compositions of diets combined with water restriction, as well as hematobiochemical monitoring for a more comprehensive understanding of the effects of water restriction on animal metabolism.

## 5. Conclusions

Water supply induces changes in the physiological responses of Santa Inês crossbred ewes; however, the animals have demonstrated adaptability to water stress. Following an 80% reduction in water supply, animals exhibit mild dehydration, characterized by increased serum urea levels and decreased alanine aminotransferase activity.

It is clear that sheep adapted to the climate of semi-arid regions can tolerate up to 40% water restriction for a given period. However, it seems feasible that future studies with reduced days of confinement should be conducted to determine the best period for applying water restriction of up to 40%. Furthermore, assessment of cortisol, aldosterone, and vasopressin levels throughout the experimental period is necessary and relevant.

## Figures and Tables

**Table 1:** Ingredients and chemical composition of experimental diets.

	Ingredients	g/kg Dry Matter		
	Elephant grass	460		
	Corn meal	381		
	Soybean meal	132		
	Mineral salt ^1^	20		
	Urea	7		
Chemical composition (g/kg dry matter)				
	Elephant grass	Corn meal	Soybean meal	Diet
Dry matter ^2^	261.9	889.3	886.1	576.26
Mineral matter	105.2	12.9	64.8	61.86
Crude protein	105.5	89.9	487.4	149.13
Ether extract	28.7	45.1	19.0	32.89
Neutral detergent fiber	708.7	111.6	15.46	370.56
Acid detergent fiber	419.5	33.7	88.5	206.97
Total carbohydrates	830.5	859.9	42.8	715.30
Non-fiber carbohydrates	174.0	642.0	27.85	328.31
Total digestible nutrients	570.1	850.0	80.48	596.71

^1^ Guaranteed levels per kilo of product guaranteed by the manufacturer: calcium (min.) 190 g; phosphorus (min.) 75 g; magnesium (min.) 10 g; chlorine (min.) 218 g; sulfur (min.) 70 g; sodium (min.) 143 g; copper (min.) 300 mg; cobalt (min.) 405 mg; iron (min.) 500 mg; iodine (min.) 80 mg; manganese (min.) 1100 mg; selenium (min.) 30 mg; zinc (min.) 4600 mg; fluorine (max.) 0.87 g; solubility of phosphorus (P) in 2% citric acid (min.): 95%. ^2^ in g/kg natural matter.

**Table 2:** Average and total value of water offered during the experimental period.

Variables	Water Supplies (%)			
	100%	80%	60%	40%
∑ Water supplied (L)	355.0	112.22	88.16	64.10
$\bar{X}\;$ Water supplied (L/day)	5.00	1.58	1.24	0.903
Minimum quantity supplied (L/day)	-	0.77	0.61	0.44
Maximum quantity supplied (L/day)	-	2.46	1.97	1.48

**Table 3:** Physiological parameters and sweating rate of ewes subjected to different levels of water supply.

Variables	Water Supplies (%)				SEM	*p*-Value	
	100	80	60	40		L	Q
Respiratory rate (mov/min) ^1^	66.56	56.03	67.56	79.34	2.29	<0.001	<0.001
Heart rate (beats/min) ^2^	100.7	97.5	97.06	101.12	0.93	0.844	<0.001
Rectal temperature (°C) ^3^	39.01	38.83	38.86	38.97	0.042	0.566	<0.001
	Sweating rate (g/m^2^/h)						
Neck	74.98	77.45	78.69	128.82	35.8	0.317	0.510
Loin	56.56	63.82	83.34	64.26	8.77	0.285	0.143

SEM = standard error of the mean; *p*-value = probability value; L = linear; Q = quadratic; significant at the 5% probability level. Equation: ^1^ ŷ = 146.191 − 2.202x + 0.014x^2^; R^2^ = 0.91, ^2^ ŷ = 119.368 − 0.640x + 0.0045x^2^; R^2^ = 0.98, ^3^ ŷ = 39.691 − 0.025x + 0.000184x^2^; R^2^ = 0.95.

**Table 4:** Observation times on respiratory rate (RR), heart rate, rectal temperature (RT), and sweat rate of ewes subjected to different levels of water supply.

Hour	Respiratory Rate (mov/min)	Heart Rate (Beats/min)	Rectal Temperature (°C)
09h00	67.12 c	105.21 b	38.92 ab
12h00	85.37 b	111.03 a	38.97 ab
15h00	93.81 a	107.28 a	39.20 a
18h00	84.71 b	106.12 b	39.07 a
21h00	63.62 c	95.06 c	38.98 ab
00h00	49.68 d	86.46 d	38.86 b
03h00	46.25 d	89.78 cd	38.76 b
06h00	48.40 d	91.81 c	38.57 b
SEM	3.24	1.32	0.06
*p*-value	<0.001	<0.001	<0.005
	Sweating rate (g/m^2^/h)		
Hour	Neck	Loin	
09h00	116.12	62.4	
15h00	65.32	71.59	
SEM	25.31	6.20	
*p*-value	0.178	0.303	

SEM = standard error of the mean; *p*-value = probability value; ^a,b,c,d^ means followed by different letters in the line differ statistically by Tukey’s test at the 5% probability level.

**Table 5:** Biochemical parameters of ewes subjected to different levels of water supply.

Variables	Water Supplies (%)				SEM	*p*-Value	
	100	80	60	40		L	Q
Albumin (g/dL)	34.3	41.08	35.89	37.71	1.66	0.502	0.147
Creatinine (mg/dL)	1.1	1.13	1.17	1.25	0.07	0.155	0.690
Glucose (g/dL)	55.95	55.2	54.5	53.16	2.36	0.397	0.903
Urea (mg/dL) ^1^	70.91	82.5	82.75	93.75	4.40	0.002	0.948
TP (g/dL)	68.08	62.16	65.45	67.04	3.04	0.990	0.229
Cholesterol (mg/dL)	58.45	64.95	57.87	54.25	3.80	0.195	0.326
Triglycerides (mg/dL)	21.83	21.79	21.87	20.08	2.15	0.597	0.688
AST (UI/L)	47.79	62.08	50.41	64.45	5.54	0.133	0.982
GGT (UI/L)	17.91	16.08	20.83	18.33	1.39	0.346	0.814
ALT (UI/L) ^2^	14.79	14.24	14.12	9.41	1.45	0.037	0.103

TP = total serum protein; AST = *aspartate aminotransferase*; GGT = *gamma-glutamyl transferase*; ALT = *alanine aminotransferase*; SEM = standard error of the mean; *p*-value = probability value; L = linear; Q = quadratic; significant at the 5% probability level. Equation: ^1^ ŷ = 106.368 + 0.341x; R^2^ = 0.89, ^2^ ŷ = 7.658 − 0.0712x; R^2^ = 0.50.

**Table 6:** Hematological parameters of ewes submitted to different levels of water supply.

Variables	Water Supplies (%)				SEM	*p*-Value	
	100	80	60	40		L	Q
WBC (cells/mm^3^)	7.66	5.83	6.96	6.63	0.64	0.507	0.258
RBC (10^6^/µL) ^1^	11.68	12.22	12.15	13.02	0.36	0.022	0.655
HGB (g/dl)	12.27	12.50	12.80	12.83	0.36	0.229	0.797
HCT (%)	36.41	37.67	38.30	38.82	1.13	0.131	0.747
MCV (fL)	31.26	31.15	31.15	30.05	0.81	0.330	0.552
MCH (Pg)	10.36	10.17	10.32	9.82	0.24	0.160	0.603
MCHC (g/dL)	33.42	33.32	33.50	32.90	0.25	0.237	0.343
RDWC (%)	18.01	18.73	18.28	17.85	0.48	0.670	0.320
PC (10^3^/µL)	577.29	698.95	660.41	634.00	73.08	0.690	0.814
MPV (fL)	3.63	3.72	4.98	3.65	0.61	0.638	0.253
PDWC (%)	15.46	15.45	15.37	15.45	0.08	0.778	0.625
PCT (%)	0.21	0.25	0.24	0.23	0.02	0.737	0.256

WBC = white blood cell count; RBC = red blood cell count; HGB = hemoglobin count; HCT = hematocrit; MCV = mean corpuscular volume; MCH = mean corpuscular hemoglobin; MCHC = mean corpuscular hemoglobin concentration; RDWC = red cell distribution width; PC = platelet count; MPV = mean platelet volume; PDWC = platelet distribution width; PCT = procalcitonin; SEM = standard error of the mean; p-value = probability value; L = linear; Q = quadratic; significant at the 5% probability level. Equation: 1 ŷ = 13.650 + 0.0196x; R2 = 0.84.

**Table 7:** Creatinine and urea concentration and sedimentoscopy frequency in the urine of ewes submitted to different water supplies.

Variables	Water Supplies (%)				SEM	*p*-Value	
	100	80	60	40		L	Q
Creatinine (mg/dL) ^1^	52.45	41.86	41.93	39.93	3.40	0.020	0.218
Urea (mg/dL)	43.25	43.33	46.08	42.58	6.00	0.978	0.768
Sedimentoscopy							
Leukocytes	0/8	1/8	0/8	1/8	-	-	-
Crystals	1/8	4/8	1/8	1/8	-	-	-
Yeast	0/8	1/8	0/8	0/8	-	-	-
	Bacteria						
Absent	3/8	0/8	0/8	1/8	-	-	-
+	3/8	4/8	4/8	2/8	-	-	-
++	1/8	2/8	1/8	1/8	-	-	-
+++	1/8	2/8	2/8	4/8	-	-	-
	Cells						
Rare	6/8	4/8	4/8	3/8	-	-	-
Moderate	0/8	3/8	2/8	2/8	-	-	-
High	2/8	1/8	2/8	3/8	-	-	-

The quantitative criteria adopted for bacteria count include absent (average count < 1 cell/field); a cross (+) (count of 1 to 3 cells/field); two crosses (++) (count of 3 to 5 cells/field); three crosses (+++) (>5 cells/field); SEM = standard error of the mean; *p*-value = probability value; L = linear; Q = quadratic; significant at the 5% probability level. Equation: ^1^ ŷ = 30.923 − 0.187 x; R^2^ = 0.72.

**Table 8:** Density, pH, protein, urobilinogen and red blood cell concentrations, color, and appearance of urine from ewes subjected to different water supplies.

Variables	Water Supply (%)				SEM	*p*-Value	
	100	80	60	40		L	Q
Density	1.005	1.006	1.006	1.008	0.012	0.058	0.627
pH ^1^	7.62	8.68	8.75	8.31	0.33	0.169	0.034
Protein ^2^	0.00	3.75	3.75	5.62	2.21	0.039	0.675
Urobilinogen ^3^	0.10	0.10	0.10	0.32	0.07	0.050	0.138
Red blood cells	0.00	0.00	0.00	1.00	0.50	0.190	0.326
Color							
Light yellow	0/8	3/8	3/8	3/8	-	-	-
Straw yellow	3/8	0/8	2/8	2/8	-	-	-
Citron yellow	5/8	5/8	3/8	3/8	-	-	-
	Appearance						
Clear	4/8	3/8	3/8	4/8	-	-	-
Cloudy	4/8	5/8	5/8	4/8	-	-	-

*p*-value = probability value; SEM = standard error of the mean; L = linear; Q = quadratic; significant at the 5% probability level. Equation: ^1^ ŷ = 4.966 + 0.121x − 0.00094x^2^, R^2^ = 0.98; ^2^ ŷ = 9.186 + 0.0844x, R^2^ = 0.85; ^3^ ŷ = 0.393 + 0.00338x, R^2^ = 0.60.

## Data Availability

The datasets generated during and/or analysed during the current study are available in the https://doi.org/10.1016/j.smallrumres.2022.106801; https://doi.org/10.1016/j.livsci.2021.104402; https://doi.org/10.1016/j.smallrumres.2023.107021.

## References

[1] Dias e Silva T.P., Torreão J.N.C., Marques C.A.T., Araújo M.J., Bezerra L.R., Dhanasekaran D.K., Sejian V. (2016). Effect of multiple stress factors (thermal, nutritional and pregnancy type) on adaptive capability of native ewes under semi-arid environment. J. Therm. Biol..

[2] Araújo G.G.L., Costa S.A.P., Moraes S.A., Queiroz M.A.A., Gois G.C., Santos N.M.S.S., Albuquerque I.R.R., Moura J.H.A., Campos F.S. (2019). Supply of water with salinity levels for Morada Nova sheep. Small Rumin. Res..

[3] Benatallah A., Ghozlane F., Marie M. (2019). The effect of water restriction on physiological and blood parameters in lactating dairy cows reared under Mediterranean climate. Asian-Australas. J. Anim. Sci..

[4] Akinmoladun O.F., Muchenje V., Fon F.N., Mpendulo C.T. (2019). Small ruminants: Farmers’ hope in a world threatened by water scarcity. Animals.

[5] Araújo C.A., Magalhães A.L.R., Araújo G.G.L., Campos F.S., Gois G.C., Santos K.C., Matos M.H.T., Nascimento D.B., Santos N.S. (2023). Correlation between mineral profile, physical-chemical characteristics, and proximate composition of meat from Santa Ines ewes under water restriction. Semin. Ciências Agrárias.

[6] Santos F.M., Araújo G.G.L., Souza L.L., Yamamoto S.M., Queiroz M.A.A., Lanna D.P.D., Moraes S.A. (2019). Impact of water restriction periods on carcass traits and meat quality of feedlot lambs in the Brazilian semi-arid region. Meat Sci..

[7] Souza L.L., Araújo G.G.L., Turco S.H.N., Moraes S.A., Voltolini T.V., Gois G.C., Campos F.S., Santos M.C.R., Santos F.M. (2022). Water restriction periods affect growth performance and nutritional status of Santa Inês sheep in the Brazilian Semi-arid. Semin. Ciências Agrárias.

[8] Nobre I.S., Araújo G.G.L., Santos E.M., Carvalho G.G.P., Albuquerque I.R.R., Oliveira J.S., Ribeiro O.L., Turco S.H.N., Gois G.C., Silva T.G.F. (2023). Cactus pear silage to mitigate the effects of an intermittent water supply for feedlot lambs: Intake, digestibility, water balance and growth performance. Ruminants.

[9] Albuquerque I.R.R., Araújo G.G.L., Voltolini T.V., Moura J.H.A., Costa R.G., Gois G.C., Costa S.A.P., Campos F.S., Queiroz M.A.A., Santos N.M.S.S. (2020). Saline water intake effects performance, digestibility, nitrogen and water balance of feedlot lambs. Anim. Prod. Sci..

[10] Adeniji Y.A., Sanni M.O., Abdoun K.A., Samara E.M., Al-Badwi M.A., Bahadi M.A., Alhidary I.A., Al-Haidary A.A. (2020). Resilience of lambs to limited water availability without compromising their production performance. Animals.

[11] Saini B.S., Kataria N., Kataria A.K., Sankhala L.N. (2013). Dehydration stress associated variations in rectal temperature, pulse and respiration rate of Marwari sheep. J. Stress Physiol. Biochem..

[12] Lee H., Romero J., IPCC (2023). Summary for policymakers. Climate change 2023: Synthesis Report. Contribution of Working Groups I, II and III to the Sixth Assessment Report of the Intergovernmental Panel on Climate Change.

[13] Silva A.S.S., Arnan X., Medeiros P.M. (2024). Climate change may alter the availability of wild food plants in the Brazilian semiarid. Reg. Environ. Change.

[14] Al-Ramamneh D., Riek A., Gerken M. (2012). Effect of water restriction on drinking behaviour and water intake in German black-head mutton sheep and Boer goats. Animal.

[15] Chedid M., Jaber L.S., Giger-Reverdin S., Duvaux-Ponter C., Hamadeh S.K. (2014). Review: Water stress in sheep raised under arid conditions. Can. J. Anim. Sci..

[16] Halfen J., Rahal N.M., Barbosa A.A., Corrêa M.N., Del Pino F.A.B., Rabassa V.R., Brauner C.C., Schmitt E. (2020). Influência da restrição alimentar e do estresse térmico sobre parâmetros fisiológicos em ovinos. Arq. Bras. Med. Vet. Zootec..

[17] Mengistu U.L., Puchala R., Sahlu T., Gipson T.A., Dawson L.J., Goetsch A.L. (2016). Comparison of different levels and lengths of restricted drinking water availability and measurement times with Katahdin sheep and Boer and Spanish goat wethers. Small Rumin. Res..

[18] Yetisgin S.O., Şen U. (2020). Resilience to drought in semi-desert sheep: Effects of water restriction during pregnancy on placental efficiency in the Awassi breed. Anim. Sci. J..

[19] Koppen W. (1923). Grundriss der Klimakunde: Outline of Climate Science.

[20] Lima P.R., Araújo C.A., Campos F.S., Menezes V.G., Ribeiro N.L., Araújo G.G.L., Menezes D.R., Matos M.H.T., Queiroz M.A.A., Santos E.M. (2023). Reductions in the water supply to crossbred Santa Inês ewes in the Brazilian semi-arid: Apparent nutrient digestibility, water and nitrogen balance, and performance. Small Rumin. Res..

[21] Araújo C.A., Magalhães A.L.R., Araújo G.G.L., Campos F.S., Gois G.C., Matos M.H.T., Queiroz M.A.A., Menezes V.G., Costa C.J.P., Santos K.C. (2021). Effect of reduced of water supply on carcass characteristics, non-carcass components and the volume of digestive compartments of Santa Inês ewes. Livest. Sci..

[22] Araújo E.J.B., Pereira F.D.S., Nunes T.S.S., Cordeiro A.E., Silva H.C., Queiroz M.A.A., Gois G.C., Rodrigues R.T.S., Menezes D.R. (2022). Nutritional value, feeding behavior, physiological parameters, and performance of crossbred Boer goats kids fed butterfly pea hay and cactus pear meal. Spanish J. Agric. Res..

[23] NRC (2007). National Research Council. Nutrient Requirements of Small Ruminants: Sheep, Goats, Cervids, and New World Camelids.

[24] Mader T.L., Davis M.S., Brown-Brandl T. (2006). Environmental factors influencing heat stress in feedlot cattle. J. Anim. Sci..

[25] Silanikove N., Koluman N. (2015). Impact of climate change on the dairy industry in temperate zones: Predications on the overall negative impact and on the positive role of dairy goats in adaptation to earth warming. Small Rumin. Res..

[26] Rosa P.R., Araújo G.G.L., Turco S.H.N., Moraes S.A., Alves J.N., Gois G.C., Santos R.D., Campos F.S. (2019). Ingestive behavior and physiological parameters of sindhi heifers receiving saline water. J. Agric. Sci..

[27] Maia A.S.C., Silva R.G., Loureiro C.M.B. (2005). Sensible and latent heat loss from the body surface of Holstein cows in a tropical environment. Int. J. Biomet..

[28] Garcia-Navarro C.E.K. (2005). Manual de Urinálise Veterinária.

[29] Santarosa B.P., Ferreira D.O.L., Rodrigues M.M.P., Dantas G.N., Sacco S.R., Lopes R.S., Dias A., Gonçalves R.C. (2016). Avaliação clínica, laboratorial e anatomopatológica do sistema urinário de ovinos confinados com ou sem suplementação de cloreto de amônio. Pesq. Vet. Bras..

[30] Taffarel L.E., Costa P.B., Pozza M.S.S., Wobeto J.R., München E.P. (2012). Correlação entre características físicas, pH e contagem bacteriana da urina de ovinos. Synerg. Scyent..

[31] SAS University (2015). Sas/Stat University User Guide.

[32] Hoffmann G., Herbut P., Pinto S., Heinicke J., Kuhla B., Amon T. (2020). Animal-related, non-invasive indicators for determining heat stress in dairy cows. Biosyst Eng..

[33] Slimen I.B., Chniter M., Najar T., Ghram A. (2019). Meta-analysis of some physiologic, metabolic and oxidative responses of sheep exposed to environmental heat stress. Livest. Sci..

[34] McKinley M., Trevaks D., Weissenborn F., McAllen R. (2017). Interaction between thermoregulation and osmoregulation in domestic animals. Rev. Bras. Zootec..

[35] West J.W. (2003). Effects of heat-stress on production in dairy Cattle. J. Dairy Sci..

[36] Maia A.S.C., Nascimento S.T., Nascimento C.C.N., Gebremedhin K.G. (2016). Thermal equilibrium of goats. J. Therm. Biol..

[37] Mendes A.M.P., Azevedo M., Lopes P.M.O., Moura G.B.A. (2014). Zoneamento bioclimático para a raça ovina Dorper no Estado de Pernambuco. Pesq. Agropec. Bras..

[38] Silva E.M.N., Souza B.B., Sousa O.B., Silva G.A.S., Freitas M.M.S. (2010). Avaliação da adaptabilidade de caprinos ao semiárido através de parâmetros fisiológicos e estruturas do tegumento. Rev. Caat..

[39] Reece W.O. (2017). Dukes-Fisiologia dos Animais Domésticos.

[40] De K., Kumar D., Singh K.A., Kumar K., Sahoo A., Naqvi K.M. (2015). Resilience of Malpura ewes on water restriction and rehydration during summer under semi-arid tropical climatic conditions. Small Rumin. Res..

[41] Vieira F.M.C., Pilatti J.A., Czekoski Z.M.W., Fonsêca V.F.C., Herbut P., Angrecka S., Vismara E.S., Macedo V.P., Santos M.C.R., Pasmionka I. (2021). Effect of the silvopastoral system on the thermal comfort of lambs in a subtropical climate: A preliminary study. Agriculture.

[42] McManus C.M., Lucci C.M., Maranhão A.Q., Pimentel D., Pimentel F., Paiva S.R. (2022). Response to heat stress for small ruminants: Physiological and genetic aspects. Livest. Sci..

[43] Habeeb A.A., Gad A.E., Atta M.A. (2018). Temperature-humidity indices as indicators to heat stress of climatic conditions with relation to production and reproduction of farm animals. Int. J. Biotechnol. Recent Adv..

[44] Vieira R., Louvandini H., Barcellos J., Martins C.F., McManus C. (2022). Path and logistic analysis for heat tolerance in adapted breeds of cattle in Brazil. Livest. Sci..

[45] Jawasreh K., Awaedeh F., Bani-Ismail Z., Al-Rawashdeh O., Al-Majali A. (2010). Normal hematology and selected serum biochemical values in different genetic lines of awassi Ewes in Jordan. Int. J. Vet. Med..

[46] Turner J.C. (1979). Osmotic fragility of desert bighorn sheep red blood cells. Comp. Biochem. Physiol. Part A Physiol..

[47] Getahun D., Alemneh T., Akeberegn D., Getabalew M., Zewdie D. (2019). Urea Metabolism and Recycling in Ruminants. Biomed. J. Scient. Techn. Res..

[48] Hailemariam S., Zhao S., He Y., Wang J. (2021). Urea transport and hydrolysis in the rumen: A review. Anim. Nutr..

[49] Casamassima D., Vizzarri F., Nardoia M., Palazzo M. (2016). The effect of water-restriction on various physiological variables in intensively reared Lacaune ewes. Vet. Med..

[50] Kaneko J.J., Harvey J.W., Bruss M.L. (2008). Clinical Biochemistry of Domestic Animals.

[51] Hamadeh S.K., Rawda N., Jaber L.S., Habre A., Said M.A., Barbour E.K. (2006). Physiological responses to water restriction in dry and lactating Awassi ewes. Livest. Sci..

[52] Tulu D., Gadissa S., Hundessa F. (2023). Impact of water stress on adaptation and performance of sheep and goat in dryland regions under climate change scenarios: A systematic review. J. Anim. Behav. Biomet..

[53] Xue B., Hong Q., Li X., Lu M., Zhou J., Yue S., Wang Z., Wang L., Peng Q., Xue B. (2021). Hepatic injury induced by dietary energy level via lipid accumulation and changed metabolites in growing semi-fine wool sheep. Front. Vet. Sci..

[54] Akinmoladun O.F., Fon F.N., Mpendulo C.T., Okoh O. (2020). Performance, heat tolerance response, and blood metabolites of water-restricted Xhosa goats supplemented with vitamin C. Transl. Anim. Sci..

[55] Noureddine T., Mebirouk-boudechiche L., Chaker-houd K., Aoun L. (2022). Effects of water stress on zootechnical physiological and blood parameters of Ouled Djellal ewes in Algeria. Egypt. J. Vet. Sci..

[56] Carro M.D., Cantalapiedra-Hijar G., Ranilla M.J., Molina-Alcaide E. (2012). Urinary excretion of purine derivatives, microbial protein synthesis, nitrogen use, and ruminal fermentation in sheep and goats fed diets of different quality. J. Anim. Sci..

[57] Ferreira F., Campos W.E., Carvalho A.U., Pires M.F.A., Martinez M.L., Silva M.V.G.B., Verneque R.S., Silva P.F. (2009). Clinical, hematological, biochemical, and hormonal parameters of cattle submitted to heat stress. Arq. Bras. Med. Vet. Zootec..

[58] Prado O.R., Arias E.I., Carrillo M.D., Hernández J.R., García A.C. (2021). Metabolic response to water shortage in an isolated feral sheep population. Austral J. Vet. Sci..

[59] Casamassima D., Pizzo R., Palazzo M., D’Alessandro A.G., Martemucci G. (2008). Effect of water restriction on productive performance and blood parameters in comisana sheep reared under intensive condition. Small Rumin. Res..

[60] Jaber L., Chedid M., Hamadeh S., Akıncı S. (2013). Water Stress in Small Ruminants. Responses of Organisms to Water Stress.

[61] Li B.T., Christopherson R.J., Cosgrove S.J. (2000). Effect of water restriction and environmental temperatures on metabolic rate and physiological parameters in sheep. Can. J. Anim. Sci..

[62] Ferreira D.O.L., Santarosa D.P., Surian S.R.S., Takahira R.K., Chiacchio S.B., Amorim R.M., Dias A., Gonçalves R.C. (2020). Low performance of vitamin C compared to ammonium chloride as an urinary acidifier in feedlot lambs. Ciênc. Anim. Bras..

